# Parameter Estimation of Non-Cooperative Composite Signals Based on Joint Time-Polarization Domain Separation

**DOI:** 10.3390/s26185958

**Published:** 2026-09-20

**Authors:** Yun Zhou, Fulai Wang, Hao Wu, Chen Pang, Yongzhen Li, Ping Wang

**Affiliations:** 1 The College of Electronic Science and Technology, National University of Defense Technology, Changsha 410073, China; zhouyun20@nudt.edu.cn (Y.Z.); pangchen@nudt.edu.cn (C.P.); liyongzhen@nudt.edu.cn (Y.L.); wangping@nudt.edu.cn (P.W.); 2 The Institute of Dataspace, Hefei 230000, China

**Keywords:** non-cooperative signal, composite signal separation, parameter estimation

## Abstract

The detection and identification process in radar is susceptible to disturbances from numerous non-cooperative jamming signals in addition to target signals. Accurately estimating the parameters of these non-cooperative signals is crucial for extracting the target signal. Addressing the current lack of effective solutions for estimating parameters of different types of uncooperative combined signals, this paper proposes a method for parameter estimation of non-cooperative composite signals based on joint time-polarization domain separation. This method combines amplitude and phase information from both the time and polarization domains of non-cooperative signals and integrates the JADE separation algorithm to achieve the separation and estimation of specific non-cooperative signal combinations. Experiments compare the separation performance of this method with other signal separation algorithms for non-cooperative jamming signal combinations. The results indicate that, at jamming-to-noise ratio of −10 dB to 15 dB, this method achieves better separation accuracy and precision for specific non-cooperative jamming combinations, while also accurately estimating the parameters of the non-cooperative jamming signals.

## 1. Introduction

Radars play an irreplaceable role in target detection and identification through their all-weather, all-time, and long-range detection capabilities [1,2]. However, with the rapid advancement of electronic systems, various complex forms of non-cooperative jamming signals have posed serious threats to the operational reliability of radar [3,4]. Non-cooperative jamming signals are signals that have a deceptive effect on radar target detection, such as active deceptive jamming signals. This type of jamming signal exploits the phase-coherent processing gain of the radar receiver to create false message that are similar to real targets, thereby misleading the radar and preventing it from detecting the actual target [5,6,7,8,9]. As non-cooperative signal techniques have continued to evolve, the original single-source signal has gradually evolved into composite signals. Non-cooperative combined signals are characterized by its complex structure, flexible configuration, and difficulty in identification, therefore, suppressing non-cooperative composite signals poses a major challenge in radar interference mitigation [10,11,12]. However, most existing non-cooperative signal suppression techniques require prior knowledge of signal parameters and other information. Therefore, accurately identifying the type of non-cooperative composite signal and estimating its key parameters is of great significance [13,14,15].

Active non-cooperative signal identification primarily involves classifying signal types, a topic that has been extensively studied by researchers both domestically and internationally. Driven in particular by the advantages of deep learning, significant progress has been made in both single-signal identification and non-cooperative combined signal identification [16,17,18,19,20]. However, for suppressing non-cooperative composite signals, simply identifying the type of signal is still insufficient. For example, suppression methods such as waveform design, non-matched filter design, and signal reconstruction and cancellation all require signal parameters as prior information [21,22,23,24]. Therefore, it is particularly important to perform further parameter estimation for non-cooperative signals.

Nowadays, parameter estimation in non-cooperative signals has also been extensively investigated. Among these, intermittent sampling signal and spread-spectrum signal are the classic type of non-cooperative signal. For parameter estimation of intermittent sampling signal, methods such as truncated matched filtering, dual-window edge detection, the Hilbert transform and deconvolution can be employed; these methods transform the problem of parameter estimation under intermittent sampling signal into a peak-search problem [25,26,27]. To address composite forms of intermittent sampling signals, some studies have employed Gaussian multi-peak fitting to decouple these non-cooperative composite signal forms, and then estimated the specific parameters one by one [28]. Based on the concept of composite signal separation, Lv et al. [29] designed an intermittent sampling composite signal separation network with an encoder–decoder architecture using deep learning. Wu et al. [30] adopted the YOLOv8 detection architecture to implement the separation and estimation of intermittent sampling signal composites. But these studies focus on composite signals that are combinations of signals of the same type but with different parameters, rather than combinations of jamming signals of different types. For spread-spectrum signal, existing parameter estimation methods primarily include: the combination of the autocorrelation method and the kurtosis maximization criterion, the cooperative optimization of the maximum entropy method and genetic algorithms and the joint estimation of time-domain and polarization-domain features, etc. [31,32,33,34]. However, reference [33] uses the standard JADE algorithm in a single-jamming scenario, it is difficult to apply to combinations of non-cooperative signal from different systems.

Parameter estimation for non-cooperative composite signals remains a challenging problem in radar electronic warfare. Although current research on non-cooperative signal parameter estimation is gradually shifting toward composite forms, the effectiveness and stability of these methods are difficult to guarantee. Since non-cooperative composite signals are fully superimposed in the time domain and the characteristics of the various sub-signals are completely intermixed, it is inherently difficult to estimate the parameters of composite signals directly [35]. While deep learning can achieve time-frequency separation of composite signals by constructing a separation network, this approach requires a large number of data samples for pre-training, making it difficult to ensure real-time performance in dynamic battlefield environments [36]. Traditional blind source separation (BSS) algorithms can separate two superimposed signals, such as the denoising CMN algorithm described in reference [37], but they struggle under low jamming-to-noise ratio conditions due to the degradation of statistical independence assumptions [38,39,40].

To address these difficulties and challenges, this paper proposes an improved method for estimating the parameters of non-cooperative composite signal based on polarization-extended separation. Building upon time-domain separation, this method uses an improved JADE algorithm optimized for complex-valued signals, with comprehensive validation across multiple scenarios. Simultaneously, it utilizes differences in the polarization states of various non-cooperative signals and employs the dual-polarization reception channels. It jointly analyzes information about composite signal in the time-polarization domain and utilizes the Joint Approximate Diagonalization of Eigen-matrices (JADE) algorithm to achieve separation of the composite signal, subsequently estimating the specific parameters of each isolated non-cooperative signal one by one. Experiments have demonstrated that, under polarization-expanded conditions, this method remains effective for the separation of composite signals and parameter estimation even under low jamming-to-noise ratio (JNR) conditions, and both the accuracy and robustness of the separation have been significantly improved.

The main contributions and innovations of our work are as follows:

- For non-cooperative signal combinations originating from different systems, we incorporate polarization domain information alongside time-domain separation. By exploiting the differences in polarization characteristics among various signals, we enhance the separability of signal combinations that were previously difficult to distinguish;
- By integrating an improved JADE algorithm with refinements in fourth-order cumulant computation and joint diagonalization, our method enhances separation accuracy under low JNR conditions.
- This method not only achieves the separation of non-cooperative combined jamming signals but also enables parameter estimation of the separated jamming signals.

## 2. Signal Combination Models and Separation Methods

This section constructs non-cooperative signal combination models based on all possible pairs of the three non-cooperative signals: intermittent sampling direct forwarding jamming signal (ISDJ), comb spectrum jamming signal (COMB) and spectrum spread jamming signal (SMSP). It also provides a theoretical analysis of methods for separating polarization-stretched signals. The three types of signals selected above are typical examples of false target signals.

### 2.1. Signal Combination Model

**Figure 1:**
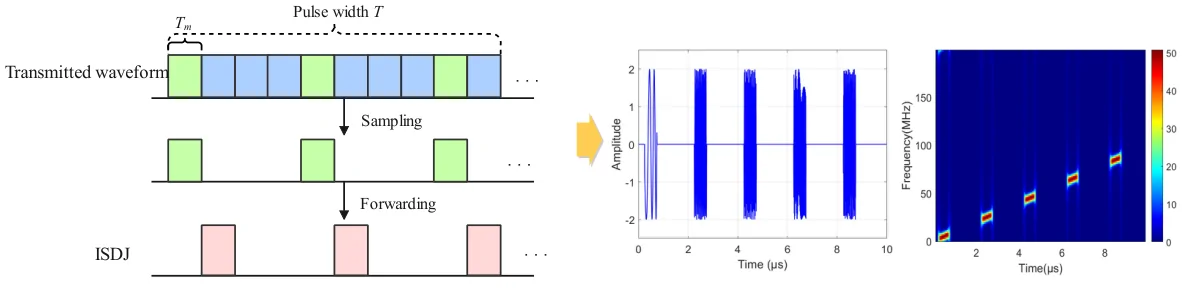
The Mechanism and signal simulation of ISDJ.

Assuming that the radar transmits a linear frequency-modulated (LFM) signal, an intermittent sampling direct forwarding jamming signal involves sampling and relaying multiple pulse cycles of the intercepted radar transmission waveform [41]. Intermittent sampling direct forwarding jamming is a type of signal that involves sampling, processing, and relaying in real time. Its mechanism and signal simulation are shown in Figure 1.

(1) $$J_{\mathrm{ISDJ}}\left(t\right)=\sum_{n=0}^{N-1}\mathrm{rect}\left(\frac{t-\tau_{J}-nT_{s}-T_{m}}{T_{m}}\right)\cdot S_{\mathrm{LFM}}\left(t-\tau_{J}-T_{m}\right)$$

Among these, $\tau_{J}$ represents the forwarding delay, $T_{m}$ denotes the sampling pulse width, $T_{s}$ indicates the sampling period, and $S_{\mathrm{LFM}}$ refers to the LFM signal transmitted by the radar.

**Figure 2:**
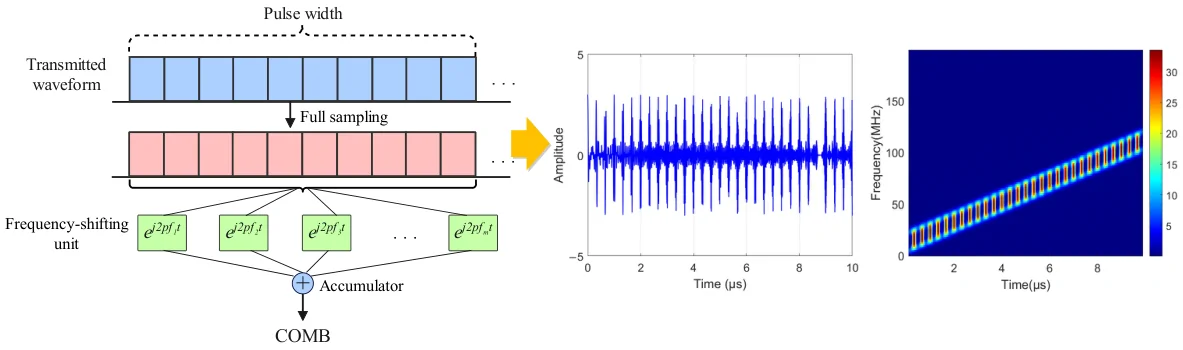
The Mechanism and signal simulation of COMB.

A comb spectrum jamming signal involves fully sampling the intercepted radar signal and then multiplying the intercepted radar signal by a comb-like signal, which is essentially a form of frequency-shift signal. Comb-like signal is the weighted sum of multiple single-frequency continuous waves and has the following signal form:

(2) $$x_{\mathrm{COMB}}\left(t\right)=\sum_{m=1}^{M}a_{m}\exp\left(j2\pi f_{m}t\right)$$

where *M* denotes the number of comb teeth, $a_{m}$ is the amplitude of the *m* signal, and fm is the frequency of the *m* signal. The comb-like signal is multiplied by the captured radar waveform to generate comb spectrum signal, which is then processed through a matched filter to produce *M* false target peaks. When $f_{m}>0$, the pulse pressure peak of the *m* component occurs ahead of the true target pulse pressure peak, creating the effect of a false target. Its model mechanism and signal simulation are shown in Figure 2.

(3) $$J_{\mathrm{COMB}}\left(t\right)=\sum_{m=1}^{M}a_{m}\mathrm{rect}\left(\frac{t}{T}\right)\exp\left[j2\pi \left(f_{0}+f_{m}\right)t+j\pi \mu t^{2}\right]$$

Here, *T* represents the pulse duration, $f_{0}$ is the carrier frequency of the transmitted waveform, and μ is the modulation frequency of the LFM signal transmitted by the radar.

**Figure 3:**
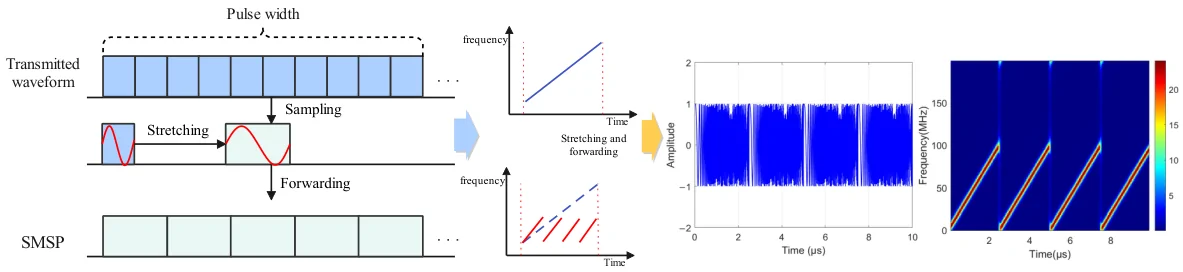
The Mechanism and signal simulation of SMSP.

A spectrum spread jamming signal occurs when the source intercepts the radar transmission signal, samples a segment of it, and retransmits it *N* times, while setting the frequency of the sampled segment to *N* times that of the original signal. Based on the principle of spectrum spread jamming, the model expression is shown in Figure 3:

(4) $$\left\{\begin{array}{c} J_{\mathrm{SMSP}}\left(t\right)=\sum_{i=0}^{N-1}J_{1}\left(t-i\frac{T}{N}\right)=J_{1}\left(t\right)⊗\sum_{i=0}^{N-1}\delta \left(t-i\frac{T}{N}\right)\\ J_{1}\left(t\right)=\exp\left(j\pi K^{\prime }t^{2}\right)\\ K^{\prime }=NK,0\leq t\leq \frac{T}{N} \end{array}\right.$$

In Equation (4), $J_{1}\left(t\right)$ represents the signal segment with the frequency modulation slope of $K^{\prime }$, where $K^{\prime }$ is *N* times the frequency modulation slope *K* of the transmitted waveform, ⊗ denotes the convolution operation.

Based on the above signal patterns, various non-cooperative jamming signals are superimposed in the time domain through space-time joint processing, ultimately forming combined jamming, and their expressions are as follows:

(5) $$J_{\mathrm{ISDJ}+\mathrm{COMB}}\left(t\right)=J_{\mathrm{ISDJ}}\left(t-\tau_{1}\right)+J_{\mathrm{COMB}}\left(t-\tau_{2}\right)+n\left(t\right)$$

(6) $$J_{\mathrm{ISDJ}+\mathrm{SMSP}}\left(t\right)=J_{\mathrm{ISDJ}}\left(t-\tau_{1}\right)+J_{\mathrm{SMSP}}\left(t-\tau_{2}\right)+n\left(t\right)$$

(7) $$J_{\mathrm{SMSP}+\mathrm{COMB}}\left(t\right)=J_{\mathrm{SMSP}}\left(t-\tau_{1}\right)+J_{\mathrm{COMB}}\left(t-\tau_{2}\right)+n\left(t\right)$$

Equations (5) to (7) describe three typical types of composite signal. Among these, $J_{\mathrm{ISDJ}+\mathrm{COMB}}$ represents the combination of ISDJ and COMB signal. $J_{\mathrm{ISDJ}+\mathrm{SMSP}}$ represents the combination of ISDJ and SMSP signal. $J_{\mathrm{COMB}+\mathrm{SMSP}}$ represents the combination of COMB and SMSP signal. $\tau_{1}$,$\tau_{2}$ represent different delays. $n\left(t\right)$ is noise.

### 2.2. Polarization-Expanded Composite Signal Separation Method

This section provides the theoretical analysis of the combined jamming separation method for polarization expansion. This method requires a dual-polarization antenna receiving system, which uses a primary antenna and a auxiliary antenna to transmit and receive signals. The polarization of the auxiliary antenna is orthogonal to that of the primary antenna, and the system operates by having the primary antenna transmit signals while both the primary and auxiliary antennas receive them simultaneously.

Define the polarization vector of the main antenna as $h_{m}$, the polarization vector of the secondary antenna is $h_{s}$, both are normalized and orthogonal to each other.

(8) $$h_{m}^{H}h_{m}=h_{s}^{H}h_{s}=1,\;h_{m}^{H}h_{s}=0$$

The polarization vectors of the two sub-signals in the composite signal are $h_{j1}$, $h_{j2}$. They can be expressed as

(9) $$h_{j1}=\left[\begin{array}{c} \cos\gamma_{1}\\ \sin\gamma_{1}e^{j\phi_{1}} \end{array}\right],h_{j2}=\left[\begin{array}{c} \cos\gamma_{2}\\ \sin\gamma_{2}e^{j\phi_{2}} \end{array}\right]$$

Here, $\gamma_{1}$, $\gamma_{2}$ are the polarization angle. $\phi_{1}$, $\phi_{2}$ are the polarization phase difference.

According to the aforementioned polarization states, the received signals from the main antenna and the auxiliary antenna can be expressed as follows:

(10) $$v_{m}\left(t\right)=h_{m}^{H}h_{j1}A_{j1}j_{1}\left(t\right)+h_{m}^{H}h_{j2}A_{j2}j_{2}\left(t\right)+n_{m}\left(t\right)$$

(11) $$v_{s}\left(t\right)=h_{s}^{H}h_{j1}A_{j1}j_{1}\left(t\right)+h_{s}^{H}h_{j2}A_{j2}j_{2}\left(t\right)+n_{s}\left(t\right)$$

Here, $j_{1}\left(t\right)$ and $j_{2}\left(t\right)$ represent two subtypes of jamming within active combined jamming. $v_{m}\left(t\right)$ and $v_{s}\left(t\right)$ represent the received signals from the main and auxiliary antennas, respectively, while $A_{j1}$ and $A_{j2}$ represent the amplitudes of the two types of jamming, $n_{m}\left(t\right)$ and $n_{s}\left(t\right)$ represent the noise in the two channels, respectively.

Therefore, Equations (10) and (11) can be written in matrix form:

(12) $$X\left(t\right)=\mathrm{AS}\left(t\right)+N\left(t\right)$$

Among these,

(13) $$X\left(t\right)=\left[\begin{array}{c} v_{m}\left(t\right)\\ v_{s}\left(t\right) \end{array}\right],S\left(t\right)=\left[\begin{array}{c} j_{1}\left(t\right)\\ j_{2}\left(t\right) \end{array}\right],N\left(t\right)=\left[\begin{array}{c} n_{m}\left(t\right)\\ n_{s}\left(t\right) \end{array}\right]$$

The expression for matrix A is

(14) $$A=\left[\begin{array}{cc} a_{11} & a_{12}\\ a_{21} & a_{22} \end{array}\right]=\left[\begin{array}{cc} h_{m}^{H}h_{j1}A_{j1} & h_{m}^{H}h_{j2}A_{j2}\\ h_{s}^{H}h_{j1}A_{j1} & h_{s}^{H}h_{j2}A_{j2} \end{array}\right]$$

To simplify the estimation of the mixing matrix in blind source separation and remove the second-order correlation in observation, the observed signals are whitened to transform them into the variance-normalized orthogonal space. Perform an eigenvalue decomposition on the observation signal $X\left(t\right)$.

(15) $$\left\{\begin{array}{c} \tilde{X}\left(t\right)=X\left(t\right)-E\left[X\left(t\right)\right]\\ R_{xx}=E\left[\tilde{X}\left(t\right){\tilde{X}}^{H}\left(t\right)\right]\\ R_{xx}=\mathrm{UD}U^{H} \end{array}\right.$$

Here, $\tilde{X}\left(t\right)$ represents the data after being mean-centered. $U=\left[u_{1},u_{2},\cdots ,u_{m}\right]$ denotes the matrix of eigenvectors, and $D=\mathrm{diag}\left(\lambda_{1},\lambda_{2},\cdots ,\lambda_{m}\right)$ denotes the diagonal matrix of eigenvalues. Furthermore, the eigenvalues are ordered as $\lambda_{1}\geq \lambda_{2}\geq \cdots \geq \lambda_{m}$.

Let the number of sub-jamming be *K*. Let $U_{K}$ be the set of eigenvectors corresponding to the first *K* largest eigenvalues, let $D_{K}=\mathrm{diag}\left(\lambda_{1},\lambda_{2},\cdots ,\lambda_{K}\right)$ be the set of corresponding eigenvalues. Then the whitening matrix is given by:

(16) $$W=D_{K}^{-1/2}U_{K}^{H}$$

Therefore, the observed signal after whitening is

(17) $$Z\left(t\right)=W\tilde{X}\left(t\right)=\mathrm{WAS}\left(t\right)+\mathrm{WN}\left(t\right)$$

The active combined jamming studied in this paper consists of two types of active jamming. Thus, K=2, the observed signal after whitening is expressed as $Z\left(t\right)={\left[z_{1}\left(t\right),z_{2}\left(t\right)\right]}^{T}$. The dimension of the fourth-order cumulative sum matrix $F_{p,q}$ is 2×2, and its (i,j) element is

(18) $${\left[F_{p,q}\right]}_{ij}=\mathrm{cum}\left(z_{i},z_{p}^{*},z_{j},z_{q}^{*}\right),i,j,p,q=1,2$$

The expansion of the fourth-order cumulative quantity is

(19) $$\begin{array}{cc} \mathrm{cum}\left(z_{i},z_{p}^{*},z_{j},z_{q}^{*}\right) & =E\left[z_{i}z_{p}^{*}z_{j}z_{q}^{*}\right]-E\left[z_{i}z_{p}^{*}\right]E\left[z_{j}z_{q}^{*}\right]\\ & -E\left[z_{i}z_{j}\right]E\left[z_{p}^{*}z_{q}^{*}\right]-E\left[z_{i}z_{q}^{*}\right]E\left[z_{p}^{*}z_{j}\right] \end{array}$$

Due to the whitened signal $Z\left(t\right)$, its second-order statistics satisfy

(20) $$E\left[z_{i}z_{j}^{*}\right]=\delta_{ij},E\left[z_{i}z_{j}\right]=0$$

Therefore, substituting Equation (20) into Equation (19) yields

(21) $$\mathrm{cum}\left(z_{i},z_{p}^{*},z_{j},z_{q}^{*}\right)=E\left[z_{i}z_{p}^{*}z_{j}z_{q}^{*}\right]-\delta_{ip}\delta_{jq}-\delta_{iq}\delta_{jp}$$

When p=1,q=1, the explicit expression for the fourth-order cumulative matrix is

(22) $${\left[F_{1,1}\right]}_{ij}=E\left[z_{i}z_{1}^{*}z_{j}z_{1}^{*}\right]-\delta_{i1}\delta_{j1}-\delta_{i1}\delta_{j1}=E\left[z_{i}z_{1}^{*}z_{j}z_{1}^{*}\right]-2\delta_{i1}\delta_{j1}$$

The corresponding elements are calculated as follows:

(23) $$\begin{array}{cc} {\left[F_{1,1}\right]}_{11} & =E\left[z_{1}^{*}z_{1}^{*}z_{1}z_{1}^{*}\right]-2=E\left[|z_{1}{|}^{4}\right]-2\\ {\left[F_{1,1}\right]}_{12} & =E\left[z_{1}^{*}z_{1}^{*}z_{2}z_{1}^{*}\right]-0=E\left[|z_{1}{|}^{2}z_{2}z_{1}^{*}\right]\\ {\left[F_{1,1}\right]}_{21} & =E\left[z_{2}^{*}z_{1}^{*}z_{1}z_{1}^{*}\right]-0=E\left[z_{2}{\left|z_{1}\right|}^{2}z_{1}^{*}\right]\\ {\left[F_{1,1}\right]}_{22} & =E\left[z_{2}^{*}z_{1}^{*}z_{2}z_{1}^{*}\right]-0=E\left[|z_{2}{|}^{2}{\left|z_{1}\right|}^{2}\right] \end{array}$$

Therefore, the $F_{1,1}$ matrix is expressed as

(24) $$F_{1,1}=\left[\begin{array}{cc} E\left[{\left|z_{1}\right|}^{4}\right]-2 & E\left[{\left|z_{1}\right|}^{2}z_{2}z_{1}^{*}\right]\\ E\left[z_{2}{\left|z_{1}\right|}^{2}z_{1}^{*}\right] & E\left[{\left|z_{1}\right|}^{2}{\left|z_{2}\right|}^{2}\right] \end{array}\right]$$

Similarly, $F_{1,2}$, $F_{2,1}$ and $F_{2,2}$ are calculated in the same way, with the expressions as follows:

(25) $$\begin{array}{cc} F_{1,2} & =\left[\begin{array}{cc} E\left[{\left|z_{1}\right|}^{2}z_{1}z_{2}^{*}\right] & E\left[{\left|z_{1}\right|}^{2}{\left|z_{2}\right|}^{2}\right]-1\\ E\left[{\left|z_{1}\right|}^{2}{\left|z_{2}\right|}^{2}\right]-1 & E\left[z_{2}z_{1}^{*}{\left|z_{2}\right|}^{2}\right] \end{array}\right]\\ F_{2,1} & =\left[\begin{array}{cc} E\left[{\left|z_{1}\right|}^{2}z_{1}z_{2}^{*}\right] & E\left[{\left|z_{1}\right|}^{2}{\left|z_{2}\right|}^{2}\right]-1\\ E\left[{\left|z_{1}\right|}^{2}{\left|z_{2}\right|}^{2}\right]-1 & E\left[{\left|z_{2}\right|}^{2}z_{2}z_{1}^{*}\right] \end{array}\right]\\ F_{2,2} & =\left[\begin{array}{cc} E\left[{\left|z_{1}\right|}^{2}{\left|z_{2}\right|}^{2}\right] & E\left[z_{1}{\left|z_{2}\right|}^{2}z_{2}^{*}\right]\\ E\left[{\left|z_{2}\right|}^{2}z_{1}z_{2}^{*}\right] & E\left[{\left|z_{2}\right|}^{4}\right]-2 \end{array}\right] \end{array}$$

In summary, the fourth-order cumulative matrix is

(26) $$F=\left\{\begin{array}{cccc} F_{1,1} & F_{1,2} & F_{2,1} & F_{2,2} \end{array}\right\}$$

To make the fourth-order cumulative matrix as diagonal as possible, we need to find the optimal unitary matrix V such that the elements of F satisfy

(27) $$V^{H}F_{p,q}V=\Lambda_{p,q}$$

where $\Lambda_{p,q}$ is a diagonal matrix.

Using the real Givens rotation method, the matrix V can be expressed as

(28) $$V=\left[\begin{array}{cc} \cos\theta & -\sin\theta \\ \sin\theta & \cos\theta \end{array}\right]$$

Perform joint diagonalization on the set of cumulative matrix $F_{p,q}$, and define the objective function as

(29) $$J\left(\theta \right)=\sum_{p=1}^{K}\sum_{q=1}^{K}\sum_{i\neq j}{\left[V^{H}F_{p,q}V\right]}_{ij}^{2}$$

Here, $J\left(\theta \right)$ denotes the sum of the squares of all off-diagonal elements, minimizing $J\left(\theta \right)$ is equivalent to maximizing the sum of the squares of the diagonal elements. For a pair of coordinates (p,q), the formula for the Givens rotation angle is

(30) $$\theta =\frac{1}{4}\arctan\left(\frac{g_{12}+g_{21}}{g_{11}+g_{22}}\right)$$

Among these,

(31) $$\begin{array}{cc} g_{11} & =\sum_{k=1}^{K^{2}}\left({\left|{\left[F_{k}\right]}_{pp}\right|}^{2}-{\left|{\left[F_{k}\right]}_{qq}\right|}^{2}\right)\\ g_{12} & =\sum_{k=1}^{K^{2}}\left({\left[F_{k}\right]}_{pp}{\left[F_{k}\right]}_{pq}-{\left[F_{k}\right]}_{qq}{\left[F_{k}\right]}_{qp}\right)\\ g_{21} & =\sum_{k=1}^{K^{2}}\left({\left[F_{k}\right]}_{pp}{\left[F_{k}\right]}_{qp}-{\left[F_{k}\right]}_{qq}{\left[F_{k}\right]}_{pq}\right)\\ g_{22} & =\sum_{k=1}^{K^{2}}\left({\left|{\left[F_{k}\right]}_{pq}\right|}^{2}-{\left|{\left[F_{k}\right]}_{qp}\right|}^{2}\right) \end{array}$$

Here, $F_{k}$ denote the *k*th fourth-order cumulative matrix, and $k=\left(p-1\right)K+q$, where the Givens rotation angle θ reflects the difference between the current unitary matrix and the idealized diagonalized matrix. The Givens rotation matrix is

(32) $$G\left(p,q,\theta \right)=\left[\begin{array}{ccccccc} 1 & \cdots & 0 & \cdots & 0 & \cdots & 0\\ \vdots & \ddots & \vdots & \; & \vdots & \; & \vdots \\ 0 & \cdots & \cos\theta & \cdots & -\sin\theta & \cdots & 0\\ \vdots & \; & \vdots & \ddots & \vdots & \; & \vdots \\ 0 & \cdots & \sin\theta & \cdots & \cos\theta & \cdots & 0\\ \vdots & \; & \vdots & \; & \vdots & \ddots & \vdots \\ 0 & \cdots & 0 & \cdots & 0 & \cdots & 1 \end{array}\right]$$

We iteratively update the V matrix using the Givens rotation matrix while simultaneously updating all accumulated matrices, and the expression for iterative updates is as follows:

(33) $$\begin{array}{cc} V & \leftarrow \mathrm{VG}\left(p,q,\theta \right)\\ F_{k} & \leftarrow G^{H}\left(p,q,\theta \right)F_{k}G\left(p,q,\theta \right),\forall k \end{array}$$

The convergence condition for the iteration is

(34) $$\max_{p,q}\left|\theta_{p,q}\right|<\varepsilon$$

Here, $\theta_{p,q}$ represents the Givens rotation angles calculated for the coordinates (p,q) in the current iteration, ε is the convergence threshold.

When the algorithm converges, the mixed signal is ultimately separated as shown in Equation (35).

(35) $$\left[\begin{array}{c} {\hat{j}}_{1}\left(t\right)\\ {\hat{j}}_{2}\left(t\right) \end{array}\right]=V^{H}Z\left(t\right)$$

**Figure 4:**
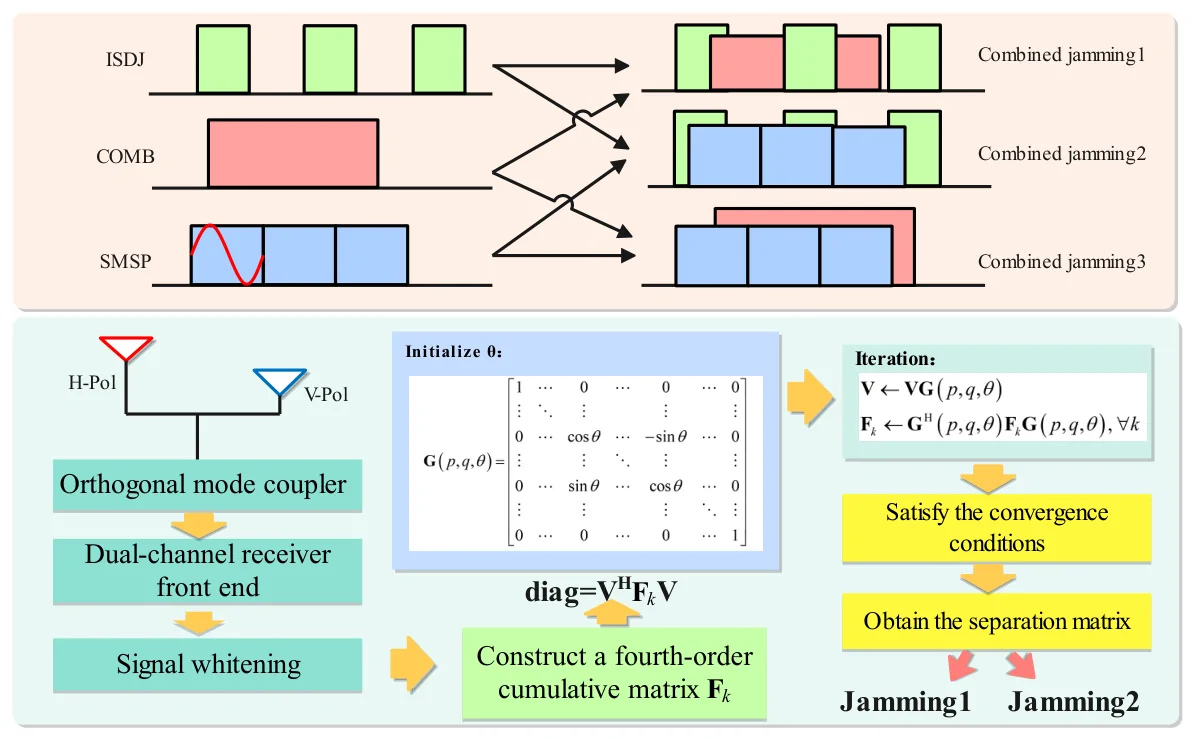
The process of active combined jamming separation methodology.

The workflow of the active combined jamming separation method with polarization expansion is shown in Figure 4. The orthogonal mode coupler is designed to enable polarization multiplexing, which involves simultaneously processing two orthogonal polarized signals while ensuring sufficient isolation between them. The above method can separate active combined jamming into pairs. By exploiting the different polarization states of the sub-jamming within the active combined jamming, the amplitude and phase states of the active combined jamming received in different polarization channels also differ, thereby amplifying the differences between the sub-jamming and improving the accuracy of jamming separation.

## 3. Parameter Estimation

This section presents the respective estimation methods of the three separated jamming types: ISDJ, COMB, and SMSP. Furthermore, it is assumed that the radar transmitted signal is the LFM waveform.

### 3.1. Parameter Estimation of ISDJ

ISDJ is generated by intercepting the radar transmission signal and relaying them with higher power through sampling [42]. Therefore, ISDJ is represented as time-domain pulse slices at the radar receiver end. For ISDJ, the dual-sliding window pulse edge detection method is adopted to transform the rising and falling edges of its pulse slices into peak values. Subsequently, peak detection is performed to locate these edges, enabling the estimation of the sampling pulse width and sampling period.

Assuming the sampling length of the interference signal is *N*, sliding window A is adjacent to sliding window B on both sides, and both have the length of *M*. Starting from the initial position of the signal, slide the moving window in increments of one sample point, calculate the mean energy in sliding window A and sliding window B ($E_{A}$ and $E_{B}$, respectively), and obtain the ratio $\xi_{AB}$ and $\xi_{BA}$.

(36) $$\xi_{AB}=E_{A}/E_{B},\xi_{BA}=E_{B}/E_{A}$$

**Figure 5:**
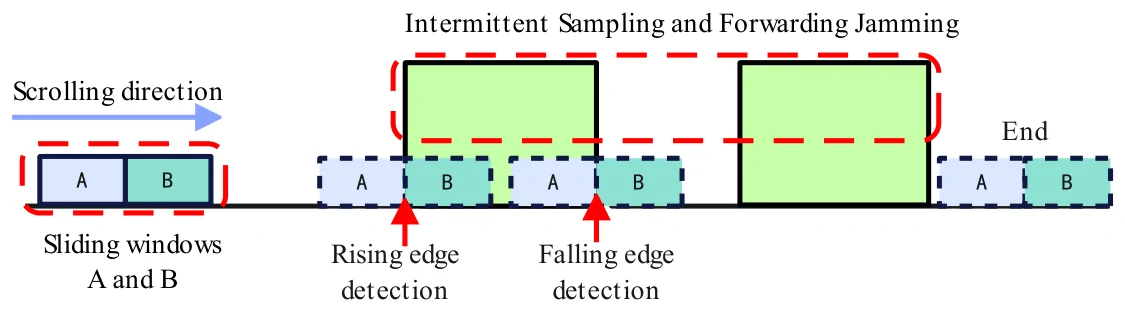
The schematic diagram of the dual-sliding window pulse edge detection method.

Figure 5 illustrates the method for detecting the rising and falling edge of the dual-sliding window pulse. When the window slides to the end of the signal, the rising-edge detection region $\zeta_{R}=\left[\xi_{BA0},\xi_{BA1},\ldots ,\xi_{BAP}\right]$ and the falling-edge detection region $\zeta_{L}=\left[\xi_{AB0},\xi_{AB1},\ldots ,\xi_{ABP}\right]$ are obtained, and P=N−2M. In the rising edge detection region $\zeta_{R}$, the peak represents the position of the rising edge, in the falling edge region $\zeta_{L}$, the peak represents the position of the falling edge. This method estimates the sampling pulse width and sampling period of the ISDJ through peak detection.

**Figure 6:**
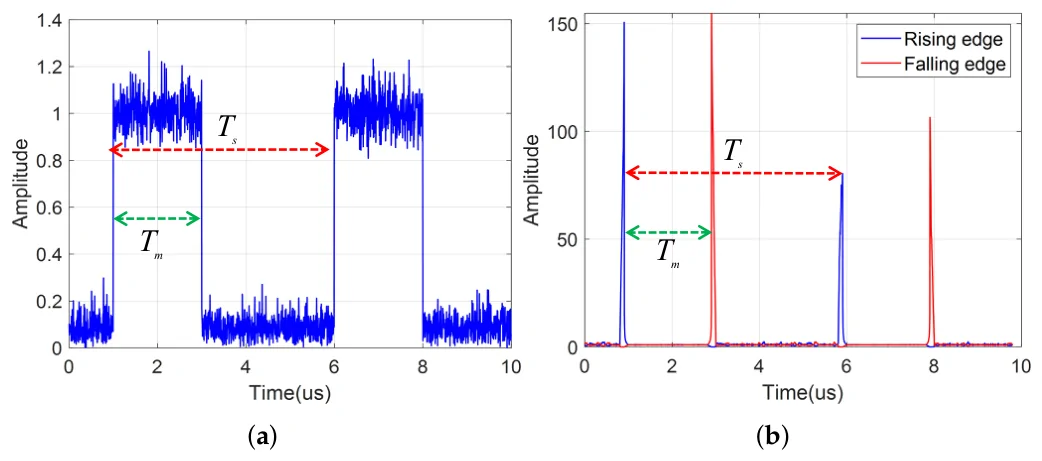
The parameters to be estimated in ISDJ: sampling period $T_{s}$ and sampling pulse width $T_{m}$. (**a**) The time-domain slicing format of ISDJ. (**b**) The peak pattern after dual-sliding window pulse edge detection.

Let the LFM waveform transmitted by the radar have the pulse width of 10 μs and the bandwidth of 100 MHz. The actual sampling pulse width of the ISDJ is 2 μs, the actual sampling period is 5 μs, and the number of sampling points in the sliding window is M=20. As shown in Figure 6, Figure 6a shows the time-domain representation of the ISDJ. Figure 6b shows the peak regions $\zeta_{R}$ and $\zeta_{L}$ of the rising and falling edges of the ISDJ. Perform peak search on regions $\zeta_{R}$ and $\zeta_{L}$. Among these, the peak value time-domain index in the rising edge region is denoted as $P_{R}=\left[p_{r1},p_{r2},\ldots ,p_{rn}\right]$, the peak value time-domain index in the falling edge region is denoted as $P_{L}=\left[p_{l1},p_{l2},\ldots ,p_{ln}\right]$. Therefore, the estimates of $T_{s}$ and $T_{m}$ are as follows:

(37) $$\begin{array}{cc} T_{s} & =\frac{1}{2\left(n-1\right)}\sum_{i=1}^{n-1}\left[\left(P_{ri+1}+P_{li+1}\right)-\left(P_{ri}+P_{li}\right)\right]\\ T_{m} & =\frac{1}{n}\sum_{i=1}^{n}\left(P_{li}-P_{ri}\right) \end{array}$$

### 3.2. Parameter Estimation of COMB

Equation (3) provides the signal model of COMB jamming. When the radar receives the COMB jamming, pulse compression is performed on it. The unit impulse response of the pulse compression is $h\left(t\right)=S^{*}\left(-t\right)$, which is the negative conjugate of the radar transmission signal. The output after matched filter is

(38) $$y_{\mathrm{COMB}\;}\left(t\right)=J_{\mathrm{COMB}\;}\left(t\right)⊗h\left(t\right)=\sum_{m=1}^{M}F_{m}\left(t,b_{m},f_{m}\right)$$

Here, ⊗ denotes convolution, and $F_{m}\left(t,b_{m},f_{m}\right)$ is the *m*th component of the radar matched filter output. The $F_{m}$ is expressed as

(39) $$\begin{array}{cc} F_{m} & =b_{m}\mathrm{sinc}\left[B\left(t-T+\frac{f_{m}}{K}\right)\left(1-\frac{\left|t-T\right|}{T}\right)\right]\\ & \cdot \exp\left\{j2\pi \left[\left(f_{0}+\frac{B}{2}+\frac{f_{m}}{2}\right)\left(t-T\right)+\frac{f_{m}}{2}T\right]\right\} \end{array}$$

In the Equation (39), $\mathrm{sinc}\left(x\right)=\sin\left(\pi x\right)/\pi x$ represents the sinc function.

According to Equations (38) and (39), the number of false targets generated after matched filtering is *M*, and each component $F_{m}$ corresponds to the single false target. Therefore, the position of the false target can be calculated using the peak position in $F_{m}$. The specific calculation is given by Equation (40).

(40) $$t_{m}=\arg\max_{t}\left(\mathrm{sinc}\left[B\left(t-T+\frac{f_{m}}{K}\right)\left(1-\frac{\left|t-T\right|}{T}\right)\right]\right)$$

**Figure 7:**
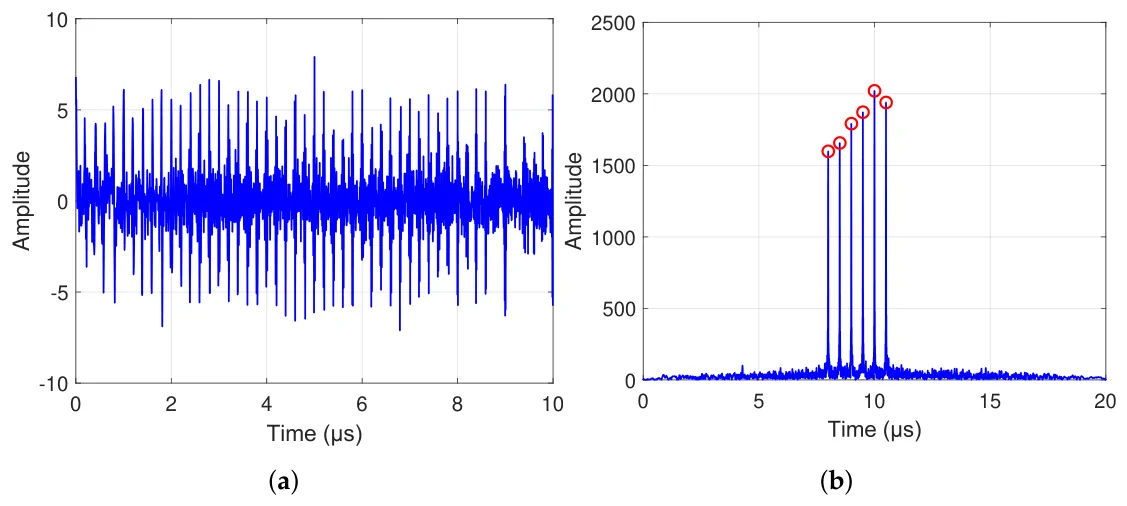
COMB jamming time-domain form and pulse compression results. (**a**) The time-domain format of COMB. (**b**) The peak pattern after pulse compression.

When $B\left(t-T+\frac{f_{m}}{K}\right)=0$, the sinc function reaches its maximum value. At this moment, the peak time position of the *m*th false target is

(41) $$t_{m}=T-\frac{f_{m}}{K}$$

where, *T* is the pulse width of the transmitted waveform and is also the center position after matched filtering. $f_{m}$ is the frequency-shifted component. *K* is the frequency modulation slope of the LFM. When $f_{m}>0$, the pre-detection false target effect occurs. The result of the peak detection after pulse compression is shown in Figure 7.

### 3.3. Parameter Estimation of SMSP

Based on the signal model of SMSP jamming in Equation (4), the time-domain deconvolution (TDC) method is used to transform the SMSP jamming. The TDC processing is a common signal processing technique. In the field of signal processing, it is used to remove or reduce the influence of the system response from the received signal, in order to better estimate the signal characteristics. SMSP jamming involves intercepting the radar transmitted signal, stretching its frequency modulation slope, and then retransmitting it. It consists of multiple signal fragments that are coherent with the radar transmitted signal. By utilizing this characteristic of SMSP, TDC processing can convert SMSP jamming into the peak signal.

Define the radar transmission signal as $S_{\mathrm{LFM}}\left(t\right)$, The jamming signal is $J_{SMSP}\left(t\right)$. The result of applying TDC processing to the jamming is

(42) $$s_{\mathrm{TDC}}\left(t\right)=\mathrm{IFFT}\left[\frac{\mathrm{FFT}\left(J_{SMSP}\left(t\right)\right)}{\mathrm{FFT}\left(S_{\mathrm{LFM}}\left(t\right)\right)}\right]$$

where, $\mathrm{FFT}\left(\cdot \right)$ stands for fast Fourier transform, and $\mathrm{IFFT}\left(\cdot \right)$ stands for inverse fast Fourier transform.

Each sub-segment of the SMSP jamming can be expressed in the form of the transmitted signal, as shown in the following expression:

(43) $$J_{1}\left(t\right)=\exp\left(j\pi NKt^{2}\right),0\leq t\leq \frac{T}{N}$$

Combining Equations (4) and (42), the expression for the TDC processing of the SMSP jamming can be written as

(44) $$s_{\mathrm{TDC}}\left(t\right)=\mathrm{IFFT}\left[\frac{\mathrm{FFT}\left(J_{1}\left(t\right)⊗\sum_{i=0}^{N-1}\delta \left(t-i\frac{T}{N}\right)\right)}{\mathrm{FFT}\left(\exp\left(j\pi Kt^{2}\right)\right)}\right]$$

**Figure 8:**
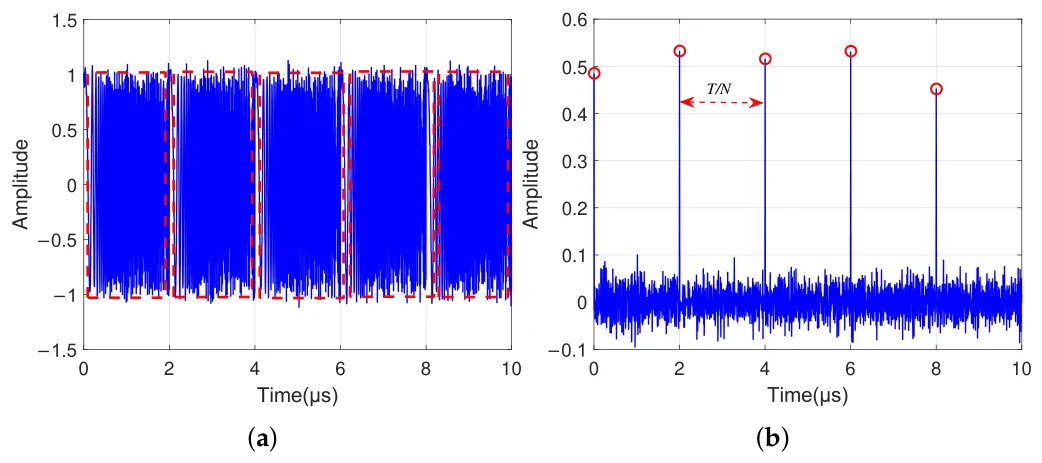
SMSP jamming and peak results after TDC processing. (**a**) The time-domain format of SMSP. (**b**) The peak pattern after TDC processing.

As can be seen in Equation (44), the process of processing SMSP jamming using TDC essentially involves multiplying the jamming signal by the radar transmission signal in the frequency domain. Since each sub-sequence of SMSP jamming is generated by frequency-modulated and rate-stretched radar transmission signals, the two exhibit strong coherence in the frequency domain. Specifically, the spectral structure of SMSP jamming contains the same frequency modulation characteristics as the radar transmission signal, with frequency shifts only existing between different sub-chips. Therefore, when the Fourier transforms of the SMSP jamming and the radar transmission signal are subtracted in the frequency domain, their common frequency components cancel each other out, while the differing components are retained. Ultimately, after the inverse Fourier transform, the jamming energy originally distributed across multiple sub-segments is compressed into a series of sharp pulse peaks. The SMSP jamming and the peak after TDC processing are shown in Figure 8.

## 4. Simulations and Results

This section presents the simulation verification of the active combined jamming parameter estimation method based on polarization-extended separation. The study primarily compared the separation accuracy and stability of polarization-extended blind source separation methods with those of conventional blind source separation methods, and also conducted the simulation analysis of the accuracy and robustness of active combined jamming parameter estimation under this method.

### 4.1. Simulation Experiment

There are many factors that influence the experimental results, including waveform and jamming parameters, as well as the initial polarization state. Therefore, detailed numerical parameters are provided to demonstrate the effectiveness of this method. All simulations are conducted in MATLAB R2024a on the computer equipped with the 2.2 GHz i9-14900HX CPU and 32 GB RAM.

The paper assumes that the radar transmit waveform is the LFM waveform with the pulse width of 10 μs and the bandwidth of 100 MHz. Assume that the jamming parameters simulate active combined jamming. The active combined jamming described in this paper is formed by pairing any two of the three types of single active jamming: ISDJ, COMB and SMSP, which is formed by the single active jamming source in the form of time-domain superposition. Furthermore, the sub-jamming can be transmitted by jammer antennas with different polarization states. Thus, the polarization states of the different sub-jamming in the active combined jamming are different. For the combination of two active jamming with different polarization states, the radar receiver uses two antennas with different polarizations to receive the signals. The main antenna uses horizontal polarization for receiving, while the auxiliary antenna uses vertical polarization for receiving. To account for non-ideal factors such as parameter uncertainty and timing jitter, disturbances are introduced. Specifically, the random Doppler offset $f_{d}$ within [−B/10,B/10] is added to the combined jamming to simulate Doppler mismatch. This paper simulates random perturbations to parameters by introducing small numerical deviations using a random function within a limited range. The specific transmission waveform and jamming simulation parameters are shown in Table 1.

Existing research has also employed deep neural networks for the estimation of active combined jamming parameters. Furthermore, lightweight neural networks coupled with FPGA acceleration have been shown to enhance recognition accuracy without sacrificing efficiency. However, network-based methods ultimately rely on data-driven training, and their dependence on data far exceeds that of traditional blind source separation algorithms. The polarization-extended separation method for estimating parameters of active combined jamming proposed in this paper does not require data pre-training, and the average runtime of this algorithm is only 9.122 s. Separation and parameter estimation can be achieved as long as the number of sub-jamming in the combined jamming is less than or equal to the number of channels. This method can still provide good performance even with a very small number of samples. Therefore, the simulation experiments in this paper are compared with traditional methods for combined jamming separation and parameter estimation in signal processing.

### 4.2. Results Analysis of Separation Method

The active combined jamming parameter estimation method using polarization expansion separation involves first separating the active combined jamming and then estimating the parameters, therefore, the performance of the combined jamming separation method directly affects the performance of the subsequent parameter estimation. Thus, we first conduct simulation verification for the polarization expansion separation method. To verify the effectiveness and stability of the active combined jamming separation method for polarization expansion, the experiment was conducted by Monte Carlo simulation. Within the jamming-to-noise ratio (JNR) range of −10 dB to 15 dB, 100 samples were generated for each type of active combined jamming at every JNR level, and each sample signal was processed using the combined jamming separation method described in this paper. Then, we analyze the separation accuracy of the polarization-expansion separation method at JNR values of −10 dB to 15 dB. JNR is defined as the ratio of the average power of the jamming signal to the average power of the noise. The reference bandwidth of JNR calculation is the radar receive bandwidth, which is typically the same as the bandwidth of the radar transmission signal.

**Figure 9:**
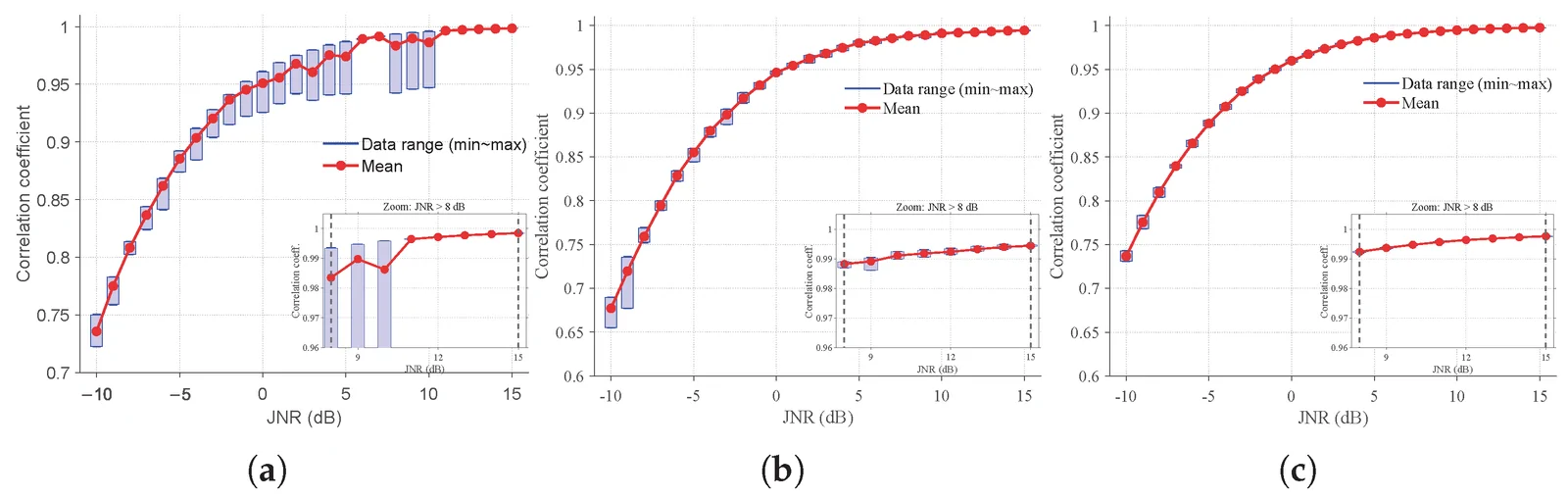
Variation of the similarity coefficient for three types of active combined interference after polarization-expanded separation under different JNR values. (**a**) Variation of the separation similarity coefficient for the ISDJ + COMB combination. (**b**) Variation of the separation similarity coefficient for the ISDJ + SMSP combination. (**c**) Variation of the separation similarity coefficient for the COMB + SMSP combination.

We introduce the similarity coefficient as the performance metric for active combined jamming separation. The expression for the similarity coefficient is shown in Equation (45). Here, $j_{1}$ and $j_{2}$ represent the two separated jamming signals, with the sampling length of N. $j_{\mathrm{ref}1}$ and $j_{\mathrm{ref}2}$ represent the original forms of the two jamming signals, which are the clean, noise-free original forms of the two jamming components before mixing and noise addition. The mean similarity between the two jamming signals after separation is used as the final evaluation metric. Figure 9 shows the variation of the similarity coefficient with JNR after active combined jamming separation.

(45) $$\mathrm{Sim}=mean(\frac{\left|\sum_{n=1}^{N}j_{1}\left(n\right)\cdot j_{\mathrm{ref}1}^{*}\left(n\right)\right|}{\sqrt{\sum_{n=1}^{N}{\left|j_{1}\left(n\right)\right|}^{2}}\cdot \sqrt{\sum_{n=1}^{N}{\left|j_{\mathrm{ref}1}\left(n\right)\right|}^{2}}}+\frac{\left|\sum_{n=1}^{N}j_{2}\left(n\right)\cdot j_{\mathrm{ref}2}^{*}\left(n\right)\right|}{\sqrt{\sum_{n=1}^{N}{\left|j_{2}\left(n\right)\right|}^{2}}\cdot \sqrt{\sum_{n=1}^{N}{\left|j_{\mathrm{ref}2}\left(n\right)\right|}^{2}}})$$

As shown in Figure 9, the figure illustrates the separation performance of the joint time-polarization domain separation method for the three jamming combinations: ISDJ + COMB, ISDJ + SMSP and COMB + SMSP. Using the average similarity coefficient as the evaluation metric for separation performance, the figure analyzes how the average similarity coefficient varies after separating these three jamming combinations across the JNR range of −10 dB to 15 dB. When the JNR value is greater than −2 dB, the similarity coefficients obtained using the active combined jamming separation method with polarization expansion are all greater than 0.9. Furthermore, when JNR > 8 dB, Sim→1, and the similarity coefficients become increasingly stable. Under conditions of low JNR, the algorithm’s separation performance deteriorates. The reason for this is that, under such conditions, the noise power is comparable to or even greater than the jamming signal power, which severely disrupts the signal’s fourth-order statistical properties and non-Gaussian nature. This leads to inaccuracies in the joint diagonalization process, thereby causing the decline in separation performance.

**Figure 10:**
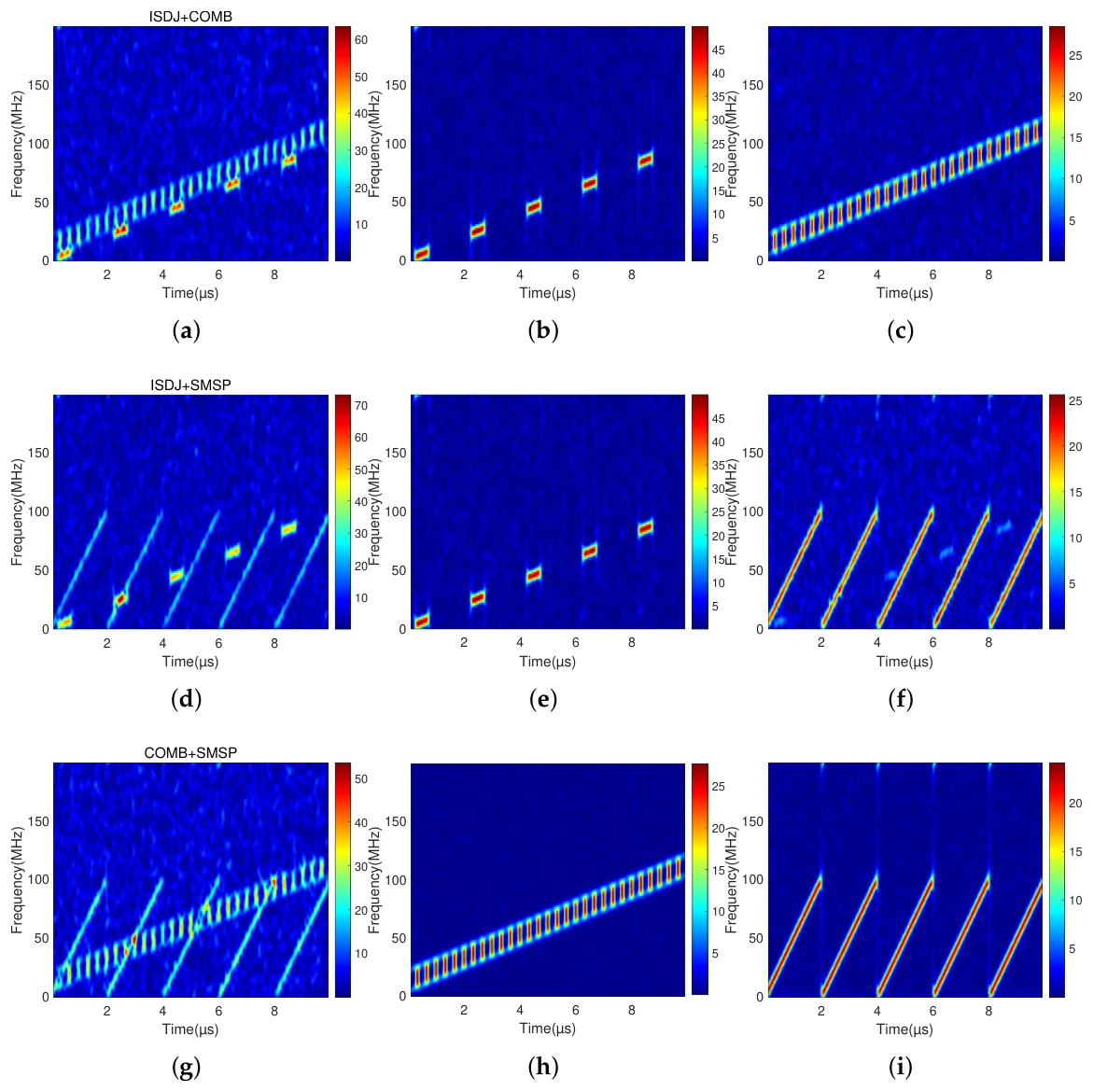
Time-frequency diagram of active combined jamming separation with polarization expansion. (**a**) Time-frequency diagram of the ISDJ + COMB jamming combination at JNR = 0 dB. (**b**) ISDJ after separation of the ISDJ + COMB jamming combination. (**c**) COMB after separation of the ISDJ + COMB jamming combination. (**d**) Time-frequency diagram of the ISDJ + SMSP jamming combination at JNR = 0 dB. (**e**) ISDJ after separation of the ISDJ + SMSP jamming combination. (**f**) SMSP after separation of the ISDJ + SMSP jamming combination. (**g**) Time-frequency diagram of the COMB + SMSP jamming combination at JNR = 0 dB. (**h**) COMB after separation of the COMB + SMSP jamming combination. (**i**) SMSP after separation of the COMB + SMSP jamming combination.

To visually demonstrate the performance of the active combined jamming separation method using polarization expansion for the three types of active combined jamming: ISDJ + COMB, ISDJ + SMSP and COMB + SMSP. Figure 10 shows the time-frequency diagrams resulting from the joint time-polarization domain separation method applied to three types of active combined jamming at the jamming-to-noise ratio of 0 dB. In Figure 10a,d,g, the time-frequency images represent ISDJ + COMB, ISDJ + SMSP and COMB + SMSP, respectively. Figure 10b,c show the time-frequency diagrams of the ISDJ and COMB jamming signals after separation of the ISDJ + COMB combination. Figure 10e,f show the time-frequency diagrams of the ISDJ and SMSP jamming signals after separation of the ISDJ + SMSP combination. Figure 10h,i show the time-frequency diagrams of the COMB and SMSP jamming signals after separation of the COMB + SMSP combination. It can be clearly seen that when JNR = 0 dB, the three types of active combined jamming are completely separated. This demonstrates that the joint time-polarization domain separation method is capable of separating the three types of combined jamming—ISDJ + COMB, ISDJ + SMSP and COMB + SMSP—and achieves good separation results.

**Figure 11:**
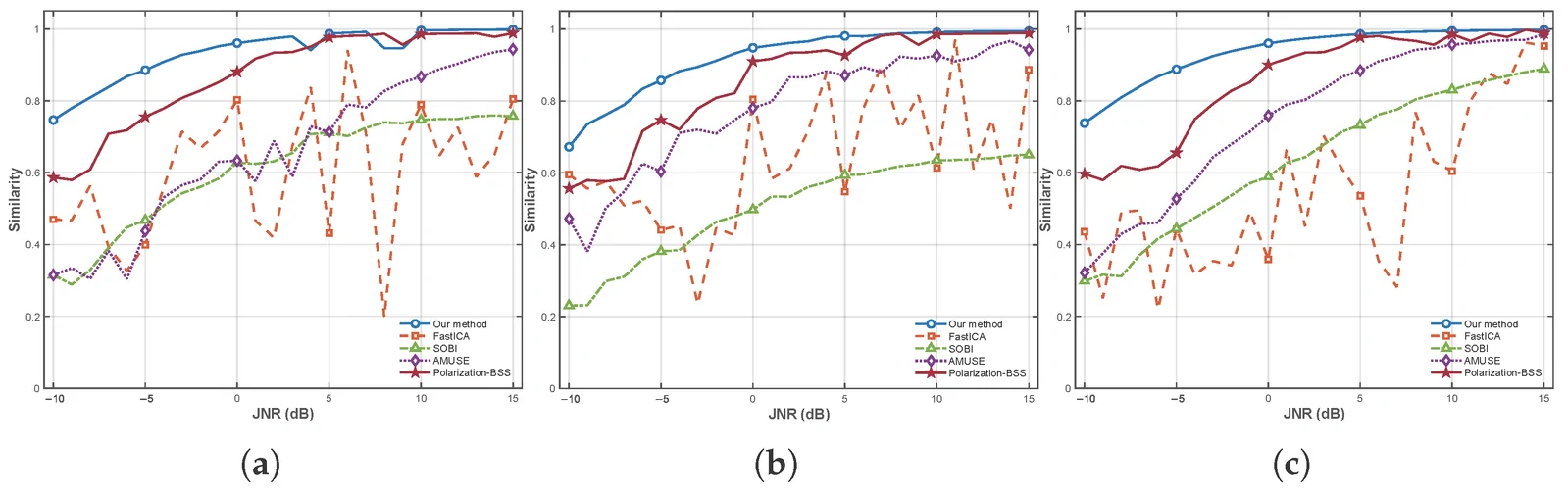
Comparison of the separation performance of different separation methods for active combined jamming. (**a**) Comparison of separation performance for ISDJ + COMB jamming combination. (**b**) Comparison of separation performance for ISDJ + SMSP jamming combination. (**c**) Comparison of separation performance for COMB + SMSP jamming combination.

Among many existing blind source separation methods, most are capable of separating active combined jamming to some extent. However, their separation performance remains limited. In the following section, simulation experiments are conducted to compare the performance differences between the proposed method and current mainstream blind source separation algorithms. Figure 11 presents the performance comparison of the method proposed in this paper with three time-domain blind source separation methods(namely FastICA, SOBI and AMUSE) and joint algorithms for dual-polarization channels(Polarization-BSS in [33]) reported in other literature. The results demonstrate the effectiveness of the polarization domain in improving the performance of combined jamming separation; furthermore, the dual-polarization channel joint algorithm proposed in this paper exhibits some advantages over other methods of the same type. Table 2 presents the average similarity coefficients for different methods under various JNR conditions (including standard deviations from multiple replicate experiments).

To provide a comprehensive comparison of the five algorithms, we analyzed their runtime and computational complexity. Among these, the method described in this paper and polarization-BSS in reference [33] use dual-polarization channels, while FastICA, SOBI and AMUSE use single-polarization channel as baseline controls. The runtime of the various algorithms is shown in Table 3. Let *K* denote the number of sources (K=2 in our simulations) and *N* denote the signal length (N≈2000). The proposed JADE-based method is dominated by the computation of fourth-order cumulant matrices, which requires $O(K^{4}\cdot N)$ operations. This is because the cumulant tensor has $K^{4}$ entries, each computed by averaging over *N* samples. *L* indicates the length of the time delay. The complexity calculations for the other methods are the same as those described above, the complexities of the various methods are shown in Table 4. Although the method described in this paper is slightly slower and more complex than the baseline method in terms of runtime and complexity, it still maintains a certain advantage when considering overall separation performance, and its average runtime is kept within 10 s, which essentially meets the time requirements of the radar operating system.

**Figure 12:**
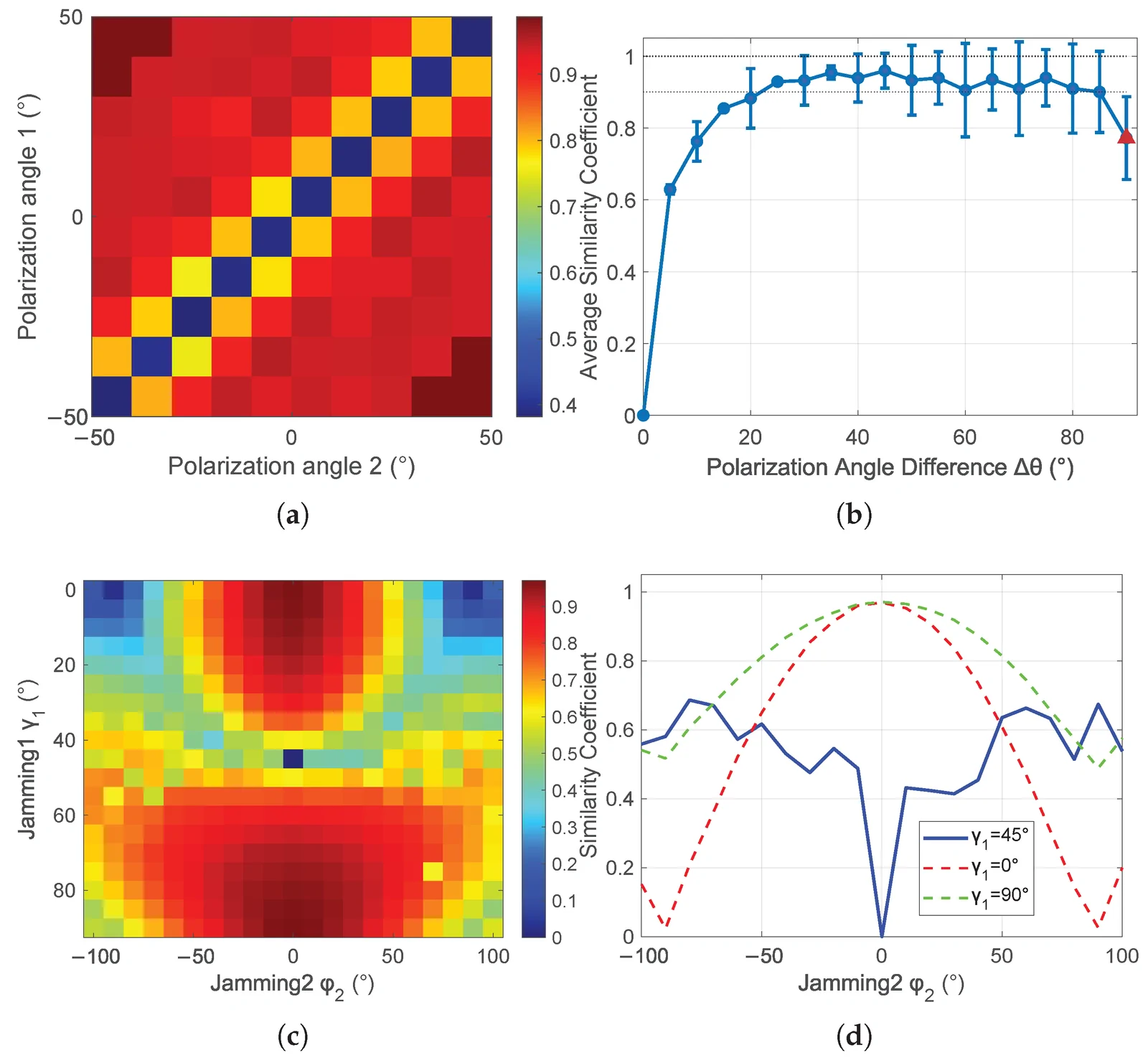
The impact of different polarization states of combined jamming on the performance of separation methods. (**a**) The impact of different line polarization angles on the separation coefficient. (**b**) The effect of polarization angle difference (The red mark indicates the angular difference is 90°). (**c**) The impact of the fully polarized state on this separation method. (**d**) Variation of the separation coefficient with phase difference at a specific polarization angle.

In practical electronic countermeasures environments, the polarization state emitted by jammers is often not the ideal linear polarization, but rather the more complex elliptical polarization or even time-varying polarization. To clarify the specific impact of polarization differences on separation performance, Figure 12 illustrates the effect of different polarization states on the jamming separation coefficient. In linear polarization jamming, when the polarization angle difference exceeds 20°, the separation coefficient can reach 0.9. Under all polarization states, including elliptical polarization, the effect of polarization differences on separation performance is more complex, the specific relationships are shown in Figure 12c,d.

### 4.3. Performance Analysis Under Non-Ideal Channel Conditions

In actual radar systems, due to antenna manufacturing errors, uneven feedline losses, and polarization rotation effects during electromagnetic wave propagation, it is often difficult to strictly achieve ideal orthogonal dual-polarization receive channels; consequently, non-ideal factors such as amplitude and phase mismatches and cross-polarization leakage (polarization coupling) are inevitable. To comprehensively evaluate the applicability of the proposed algorithm in real-world engineering environments, this section builds upon the ideal simulations described above by further introducing a channel error model to analyze the algorithm’s robustness under non-ideal conditions.

The following two types of errors are introduced to simulate non-ideal channels. Let the polarization leakage coefficient represent the degree of polarization coupling between the main channel and the auxiliary channel. Describing the frequency response inconsistency between two channels using amplitude error factors $\alpha_{m}$, $\alpha_{s}$ and phase error factors $\phi_{m},\phi_{s}$. The polarization leakage coefficient is ϵ, and $\epsilon \in \left[0,0.3\right]$. When ϵ=0, the polarization is ideally orthogonal; the larger ϵ is, the poorer the polarization isolation. Define the polarization leakage matrix as

(46) $$P_{\mathrm{leak}}=\left[\begin{array}{cc} 1 & \epsilon \\ \epsilon & 1 \end{array}\right]$$

At the same time, construct the channel mismatch matrix $H_{\mathrm{err}}=\mathrm{diag}\left[\alpha_{m}e^{j\phi_{m}},\alpha_{s}e^{j\phi_{s}}\right]$ based on the amplitude and phase errors. The resulting non-ideal mixing matrix is

(47) $$A_{\mathrm{nonideal}}=H_{\mathrm{err}}P_{\mathrm{leak}}A_{\mathrm{ideal}}$$

To realistically evaluate the degradation mechanism induced by hardware imperfections, the ideal mixing matrix is replaced by the non-ideal coupling matrix characterized by the aforementioned error parameters. In the simulation, the polarization leakage coefficient ϵ ranges from 0 to 0.3, and the amplitude-phase mismatch coefficient ranges from 0 to 0.5 (with the amplitude mismatch error and phase mismatch error in a 2:3 ratio). Under these non-ideal conditions, the resultant impact on the jamming separation coefficient is systematically investigated at representative jamming-to-noise ratios of 5 dB, 8 dB and 10 dB.

**Figure 13:**
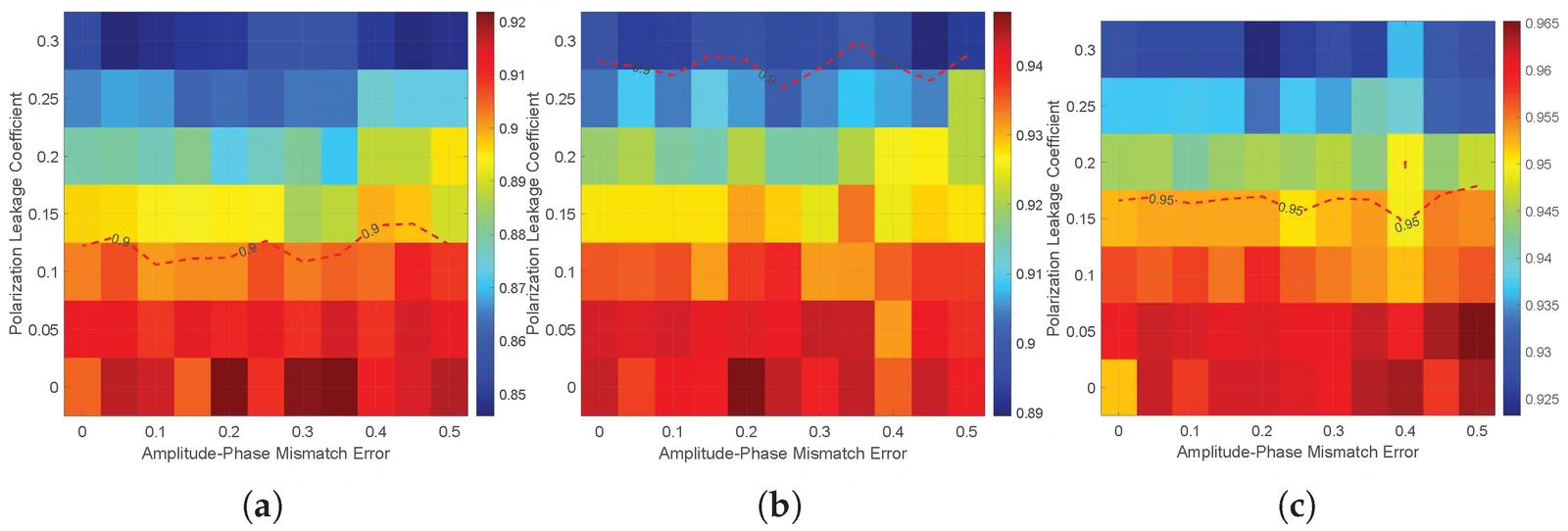
The effects of amplitude-phase mismatch and polarization leakage on the separation coefficient under different JNR. (**a**) JNR = 5 dB. (**b**) JNR = 8 dB. (**c**) JNR = 10 dB.

Figure 13 shows variations in the algorithm’s separation performance under three fixed jamming-to-noise ratio conditions, for different polarization leakage coefficients and degrees of amplitude-phase mismatch. The results show that as the polarization leakage coefficient increases, the separation performance exhibits a gradual decline. When ϵ≤0.3, the average similarity coefficient remains above 0.85, indicating that the algorithm has good tolerance for moderately polarized coupling. The simulation set contour lines with the similarity coefficient of 0.9. When the amplitude-phase mismatch coefficient varied from 0 to 0.5, the similarity coefficient did not exhibit the monotonic fluctuation, indicating that this method also has the certain degree of tolerance for the amplitude-phase mismatch coefficient.

**Figure 14:**
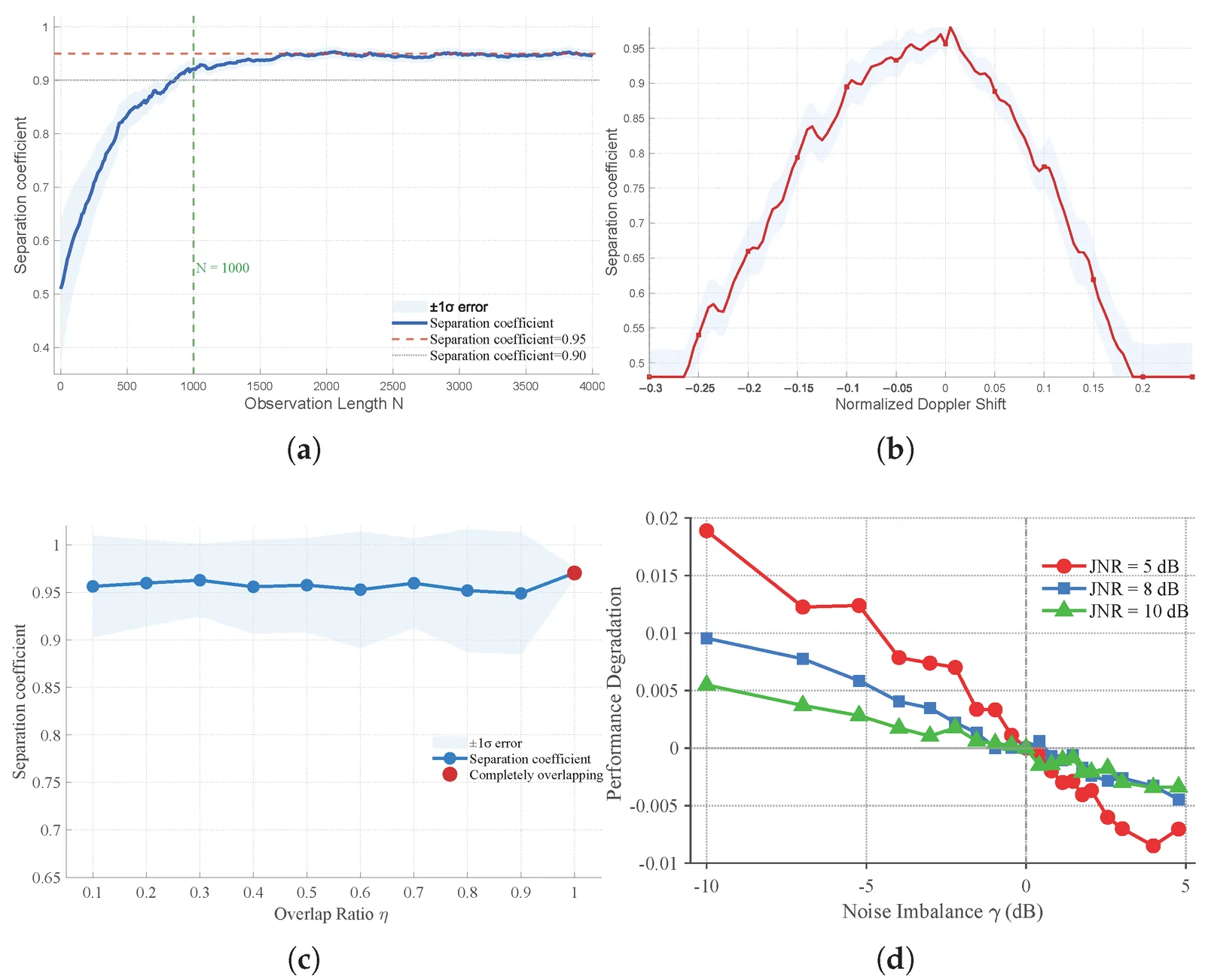
The impact of various parameter changes on algorithm performance. (**a**) Observation length. (**b**) Doppler frequency shift. (**c**) Overlap ratio of jamming (The red dot indicates complete overlap). (**d**) Noise imbalance coefficient.

In actual radar receiving systems, The parameters of signals transmitted and received by radar are often subject to uncertainty, such as the duration of timing observations and frequency shifts caused by Doppler mismatch. Figure 14a–c show simulation analyses of the algorithm’s sensitivity to observation duration, Doppler shift, and jamming overlap length, respectively. The simulation results show that this algorithm is quite sensitive to Doppler mismatch, but is not sensitive to the overlap length of jamming. Its sensitivity to the observation length depends on whether the observation length reaches a certain value, once the observation length reaches that value, the algorithm’s performance is largely unaffected by it. Furthermore, noise imbalance is also one of the non-ideal factors. $\sigma_{n_{2}}^{2}$ and $\sigma_{n_{1}}^{2}$ represent the noise power in the two channels, respectively. Specifically, we define the noise imbalance coefficient $\gamma =\sigma_{n_{2}}^{2}/\sigma_{n_{1}}^{2}$, ranging from −10 dB to 5 dB. and re-evaluate the jamming separation coefficient under the same JNR conditions (5, 8, and 10 dB). The results, presented in Figure 14d, indicate that the separation coefficient remains relatively stable. Under noise imbalances ranging from −10 dB to 5 dB, the separation coefficient fluctuates by no more than 2%. This suggests that the proposed method exhibits satisfactory robustness against moderate receiver noise imbalance.

### 4.4. Results Analysis of Parameter Estimation

The parameter estimation of active combined jamming in this paper involves estimating the parameters of the separated active jamming, the performance of the aforementioned separation method directly affects the accuracy of the parameter estimation. The performance of the proposed method in parameter estimation is evaluated by assessing the accuracy of the estimated parameters through multiple simulation experiments. It is worth noting that, due to the influence of objective factors such as signal sampling rate, the estimated results rarely match the true parameter values exactly. Therefore, in practical applications, if the difference between the estimated value and the true value falls within the system error range, the parameter value is considered to have been correctly estimated.

**Figure 15:**
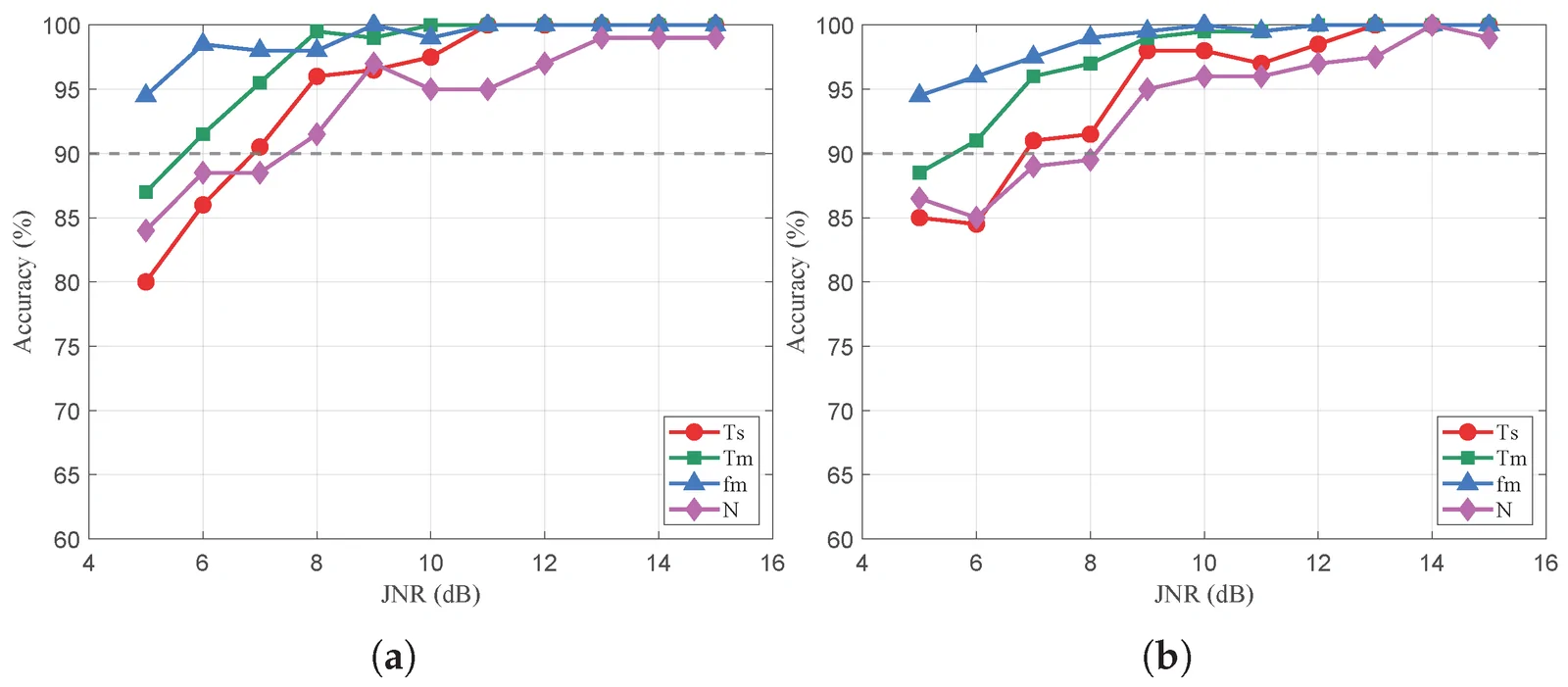
Accuracy of parameter estimation. (**a**) Estimation accuracy under the ideal channel. (**b**) Estimation accuracy when the channel polarization leakage coefficient is 0.1.

For the separated ISDJ jamming, the sampling period $T_{s}$ and sampling pulse width $T_{m}$ must be estimated; for COMB jamming, the frequency shift component $f_{m}$ must be estimated; and for SMSP jamming, the stretching coefficient *N* must be estimated. In the simulation below, we set the acceptable range for accurate estimates of $T_{s}$ and $T_{m}$ to 0μs to 0.1 μs, and the acceptable range for accurate estimates of $f_{m}$ to 0 MHz to 0.001 MHz, the estimation of the parameter *N* is considered correct only if they are comparable to the original values. These thresholds are physically derived from the system resolution limits: the time-domain tolerance of 0.1μs corresponds to ten sampling intervals ($f_{s}=100\;\mathrm{MHz}$), ensuring the de-chirping phase error Δϕ≤π/4 and coherent integration loss below 0.5dB; the frequency tolerance of 0.001MHz is set to approximately one frequency resolution cell ($\Delta f=f_{s}/N\approx 0.9766\;\mathrm{kHz}$, with N=2048), preventing comb-spectrum misalignment. We conducted 100 Monte Carlo simulations for each JNR and analyzed the estimation performance statistically. The performance of the active combined jamming parameter estimation using polarization-expanded separation is shown in Figure 15a. Figure 15b illustrates the accuracy of parameter estimation under polarization leakage in the channel. When JNR is greater than 8 dB, the estimation accuracy for all disturbance parameters exceeded 90%.

**Figure 16:**
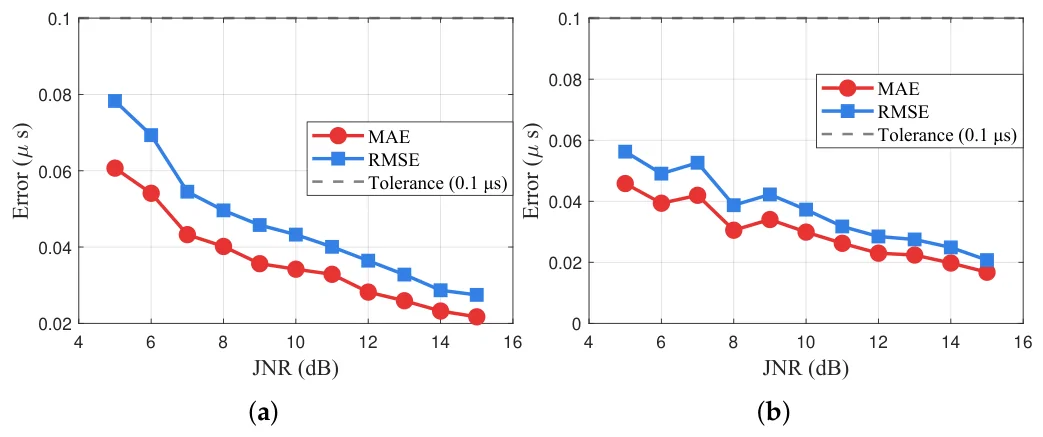

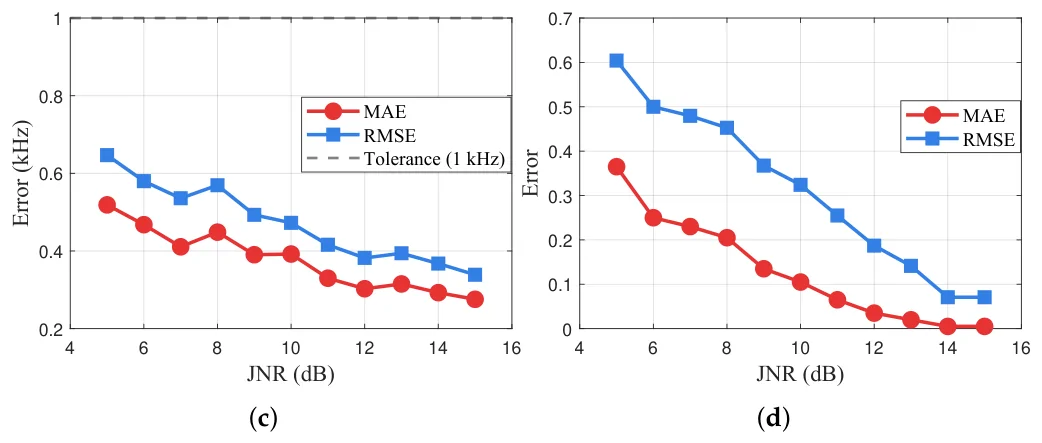
MAE and RMSE of parameter estimation under different JNR conditions. (**a**) MAE and RMSE of parameter $T_{s}$. (**b**) MAE and RMSE of parameter $T_{m}$. (**c**) MAE and RMSE of parameter $f_{m}$. (**d**) MAE and RMSE of parameter *N*.

To provide a more comprehensive and quantitative assessment of estimation accuracy, we use two standard continuous error metrics, the mean absolute error (MAE) and the root mean square error (RMSE). These metrics are defined as follows:

(48) $$\mathrm{MAE}=\frac{1}{L}\sum_{l=1}^{L}\left|{\hat{\theta }}_{l}-\theta_{\mathrm{true}}\right|$$

(49) $$\mathrm{RMSE}=\sqrt{\frac{1}{L}\sum_{l=1}^{L}{\left({\hat{\theta }}_{l}-\theta_{\mathrm{true}}\right)}^{2}}$$

where $\theta_{\mathrm{true}}$ denotes the true parameter value, ${\hat{\theta }}_{l}$ is the estimated value in the *l*-th Monte Carlo trial, and *L* is the total number of trials (L=100 in our simulations). MAE provides a measure of the average estimation bias, while RMSE is more sensitive to large errors and penalizes outliers more heavily. Figure 16 shows the changes in MAE and RMSE for the four categories of parameters. For all four parameters, both MAE and RMSE decrease monotonically as JNR increases. Furthermore, both the MAE and RMSE for the $T_{s}$, $T_{m}$ and $f_{m}$ parameters are within the error tolerance limits.

## 5. Conclusions

This paper proposes a method for estimating the parameters of the combined jamming signal through joint separation in both the time and polarization domains. The method achieves a similarity coefficient > 0.9 for all three jamming combinations when JNR > −2 dB, and parameter estimation accuracy exceeds 90% when JNR > 8 dB. The physical mechanism behind the proposed method can be explained as follows: In traditional single-polarization radar systems, the received signal is a one-dimensional mixture of all incident signals; therefore, it is impossible to separate signal sources that occupy the same time-frequency resources. By employing dual-orthogonal polarization channels, the received signal is projected into a two-dimensional polarization space, where interference sources with different polarization states become linearly separable. Subsequently, the JADE algorithm leverages the statistical independence of signals from independent physical sources to extract the underlying signal sources. Consequently, the proposed method does not rely on time-frequency diversity but instead utilizes the polarization domain as an alternative information dimension, providing a fundamentally new perspective for radar electronic protection.

This method requires a certain difference in polarization states, taking linear polarization as an example, the difference in polarization angles must be greater than 20° to achieve effective separation. The observed polarization dependence carries important practical implications. In scenarios where jamming signals exhibit similar polarization states, the proposed method may require additional polarization diversity enhancement techniques, such as dynamically adjusting the receive antenna polarization to maximize the effective polarization difference between jamming sources.

There are several promising research directions worth further exploration. First, this study assumes the presence of two interference sources, to extend this method to scenarios with three or more simultaneous jamming sources, more complex polarization channel configurations would be required, or additional information dimensions—such as spatial or frequency diversity—would need to be incorporated. In general, with *M* effective channels, up to K=M sources can be separated. Second, combining the proposed blind separation method with adaptive polarization cancellation techniquesby cascading blind and supervised strategies is expected to achieve stronger interference suppression performance. Furthermore, integrating the interference separation module with downstream target detection schemes (such as CFAR detectors) and testing them under various target polarization scattering matrix conditions is a crucial step toward optimizing the entire radar system.

## Figures and Tables

**Table 1:** Specific parameters for experimental simulation.

Category	Parameters	Value Range
LFM	Pulse width (μs)	10 (±5%)
	Bandwidth (MHz)	100 (±10%)
ISDJ	Sampling pulse width (μs)	0.8–1.2
	Sampling cycle (μs)	1.6–2.4
COMB	Number of frequency shifts	6
	Frequency interval (MHz)	4.5–5.5
	Frequency range (MHz)	−5 to 20
SMSP	Number of slices	5
	Sampling width (μs)	1.5–2.5
ISDJ + COMB	Overlap time (μs)	0 to 10
	Sampling duration (μs)	10 to 20
ISDJ + SMSP	Overlap time (μs)	0 to 10
	Sampling duration (μs)	10 to 20
COMB + SMSP	Overlap time (μs)	0 to 10
	Sampling duration (μs)	10 to 20
Jammer antenna 1	Polarization state	45° linear polarization
	Polarization vector	$\left[\frac{1}{\sqrt{2}},\frac{1}{\sqrt{2}}\right]$
Jammer antenna 2	Polarization state	−45° linear polarization
	Polarization vector	$\left[\frac{1}{\sqrt{2}},\frac{-1}{\sqrt{2}}\right]$
receiver antenna 1	Rotational disturbance	±20°
receiver antenna 2	Rotational disturbance	±20°

**Table 2:** Separation performance comparison (Similarity coefficient, mean ± std).

JNR (dB)	Similarity Coefficient (Mean ± Std)				
	**Proposed**	**FastICA**	**SOBI**	**AMUSE**	**Polarization-BSS**
−10	0.753 ± 0.087	0.482 ± 0.092	0.332 ± 0.085	0.342 ± 0.093	0.612 ± 0.102
−5	0.891 ± 0.091	0.423 ± 0.089	0.479 ± 0.098	0.435 ± 0.104	0.734 ± 0.115
0	0.942 ± 0.084	0.805 ± 0.108	0.631 ± 0.079	0.688 ± 0.086	0.876 ± 0.108
5	0.988 ± 0.052	0.431 ± 0.086	0.722 ± 0.052	0.739 ± 0.043	0.982 ± 0.056
8	0.949 ± 0.016	0.391 ± 0.092	0.756 ± 0.039	0.825 ± 0.026	0.935 ± 0.092
10	0.992 ± 0.020	0.787 ± 0.073	0.761 ± 0.099	0.868 ± 0.035	0.988 ± 0.058
15	0.993 ± 0.006	0.799 ± 0.115	0.782 ± 0.069	0.939 ± 0.053	0.984 ± 0.061

**Table 3:** Average operating time comparison.

JNR (dB)	Average Operating Time (s)				
	**Proposed**	**FastICA**	**SOBI**	**AMUSE**	**Polarization-BSS**
0	9.168	5.368	4.490	7.257	8.823
5	9.189	5.379	4.501	7.268	8.845
8	9.201	5.389	4.512	7.279	8.861

**Table 4:** Computational complexity comparison of different methods.

Method	Computational Complexity
Proposed (Joint time and polarization domains)	$O(K^{4}\cdot N)$
FastICA	$O(K^{2}\cdot N)$
SOBI	$O(K^{2}\cdot N\cdot L)$
AMUSE	$O(K^{2}\cdot N)$
Polarization-BSS	$O(K^{4}\cdot N)$

## Data Availability

The raw data supporting the conclusions of this article will be made available by the authors on request.
